# Detection of bacterial pathogens in clade E head lice collected from Niger’s refugees in Algeria

**DOI:** 10.1186/s13071-018-2930-5

**Published:** 2018-06-15

**Authors:** Meriem Louni, Nadia Amanzougaghene, Nassima Mana, Florence Fenollar, Didier Raoult, Idir Bitam, Oleg Mediannikov

**Affiliations:** 1 IRD, AP-HM, SSA, VITROME, IHU-Méditerranée Infection, Aix Marseille Univ, Marseille, France; 2 Laboratoire de Valorisation et Conservation des Ressources Biologiques (VALCOR), Faculté des Sciences, Université M’Hamed Bougara Boumerdes, Boumerdes, Algeria; 3 IRD, AP-HM, MEPHI, IHU-Méditerranée Infection, Aix-Marseille Univ, Marseille, France; 4 Laboratoire Biodiversité et Environnement: Interactions, Génomes, Département de Biologie, Université des Sciences et Technologies Houari Boumediene, Bab Ezzouar, Algeria; 5 Ecole Supérieure des Sciences de l’Aliment et des Industries Agro-Alimentaires, Algiers, Algeria

**Keywords:** *Pediculus humanus capitis*, Head lice, Niger’s refugees, Scholchildren, Migrant population, Non-migrant population, *Coxiella burnetii*, *Acinetobacter* spp., Algeria

## Abstract

**Background:**

Head lice, *Pediculus humanus capitis*, are obligate blood-sucking parasites. Phylogenetically, they occur in five divergent mitochondrial clades (A, D, B, C and E), each having a particular geographical distribution. Recent studies have revealed that head lice, as is the case of body lice, can act as a vector for louse-borne diseases. Here, we aimed to study the genetic diversity of head lice collected from Niger’s refugees (migrant population) arriving in Algeria, northern Africa, and to look for louse-borne pathogens. Comparative head lice samples collected from indigenous population of schoolchildren (non-immigrant) were also analyzed to frame the study.

**Results:**

In this study, 37 head lice samples were collected from 31 Nigerien refugees, as well as 45 head lice from 27 schoolchildren. The collection was established in three localities of eastern Algiers, north Algeria. Quantitative real-time PCR screening of pathogens bacteria and the genetic characterisation of the head lice satut were performed. Through amplification and sequencing of the *cytb* gene, results showed that all head lice of Nigerien refugees 37/82 (45.12%) belonged to clade E with the presence of four new haplotypes, while, of the 45 head lice of schoolchildren, 34/82 lice (41.46%) belonged to clade A and 11/82 (13.41%) belonged to clade B. Our study is the first to report the existence of clade E haplogroup in Nigerien head lice. DNA of *Coxiella burnetii* was detected in 3/37 (8.10%) of the head lice collected from 3 of the 31 (9.67%) migrant population. We also revealed the presence of *Acinetobacter* DNA in 20/37 (54.05%) of head lice collected from 25/31 (80.64%) of the Nigerien refugees, and in 25/45 (55.55%) head lice collected from 15/27 (55.55%) schoolchildren. All positive Nigerien-head lice for *Acinetobacter* spp. were identified as *A. baumannii*, while positive schoolchildren-head lice were identified as *A. johnsonii* 15/25 (60%), *A. variabilis* 8/25 (32%) and *A. baumannii* 2/25 (8%).

**Conclusions:**

Based on these findings from head lice collected on migrant and non-migrant population, our results show, for the first time, that head lice from Niger belong to haplogroup E, and confirm that the clade E had a west African distribution. We also detected, for the first time, the presence of *C. burnetii* and *A. baumannii* in these Nigerien head lice. Nevertheless, further studies are needed to determine whether the head lice can transmit these pathogenic bacteria from one person to another.

## Background

Sucking lice (Phthiraptera: Anoplura) are permanent parasitic insects that infest mammals, including humans [1, 2]. Two recognized genera parasitize humans, *Pthirus* and *Pediculus*, both include one species in humans, *Pthirus pubis* and *Pediculus humanus* [2, 3]. The latter comprises two ecotypes, the head louse (*Pediculus humanus capitis*) and the body louse (*Pediculus humanus humanus*). They are obligate blood-feeding parasites that thrive exclusively on human blood [4, 5]. The two ecotypes have the same life-cycle; however, they occupy distinct ecological niches and have different feeding patterns: head lice live and multiply exclusively on the scalp, while body lice live and multiply in the clothes and feed on the human body [3, 4]. Contrary to body lice, that are mostly prevalent in people living in precarious conditions where cold and lack of hygiene are present, head lice are prevalent worldwide and preferentially infest schoolchildren, regardless of social class or level of hygiene. They can cause very intense pruritus that may lead to high irritation and even wound infections [6–8]. The head louse is most likely to have been associated with humans since our pre-hominid ancestors and has been dispersed throughout the world by human migration [2].

Molecular analysis of the mitochondrial genes *cytochrome b* (*cytb*) and *cytochrome c oxidase subunit 1* (*cox*1) allowed to infer a robust phylogeographical classification of *P. humanus* into five mitochondrial clades (A, D, B, C and E), each with a particular geographical distribution [9, 10]. Clade A and D include both head and body lice, in contrast to the other clades that include only head lice, therefore the head lice encompass all the diversity [9, 10]. Clade A is the most common and has a worldwide distribution, while clade D is only found in central Africa (Ethiopia and the Democratic Republic of the Congo) [11, 12]. Clade B is confined to America, Europe, Australia, north and south Africa and was most recently reported in head lice remains from Israel, dating from approximately 2000 years ago [9, 13]. Clade C is found in Ethiopia, the Democratic Republic of Congo and in Asia (Nepal and Thailand) [11, 13–15]. Lastly, clade E, a novel clade described in west Africa (Senegal and Mali) was previously described as a sub-clade of clade C [9, 10]. Recently, this latter was also reported, for the first time, in head lice from Bobigny, France [16].

Until recently, human body lice remained the only main vector of three life-threatening infectious diseases that have killed millions of people throughout the history of humanity namely: epidemic typhus, trench fever and relapsing fever, caused by *Rickettsia prowazekii*, *Bartonella quintana* and *Borrelia recurrentis*, respectively [17]. Natural and experimental observations show that body lice may also play a role in hosting and, probably, transmitting the causative agent of plague, *Yersinia pestis*, during plague pandemics [18–20]*.* Although body lice are currently presumed to be harmful vectors of pathogens and to play a major role in all lice epidemics studied throughout human history, the status of head lice as a vector of louse-borne diseases is still under debate. Indeed, several studies report findings of body louse-borne pathogens in head lice belonging to different mitochondrial clades in different parts of the world [11, 12, 16, 21–25]. Several *Acinetobacter* species have also been detected in human head lice [11, 14, 16, 26, 27].

The emergence of humanitarian crises around the world, involving thousands of migrants, can represent an explosive risk factor in arthropod-borne disease epidemics. Indeed, several reports of louse-borne *B. recurrentis* have been from various European countries in refugee communities migrating from the Horn of Africa [28–32]. Thus, refugee populations generate a risk of the propagation of lice and their associated bacterial diseases [30]. Algeria continues to receive thousands of people from different countries, particularly from west Africa and mostly from Niger.

In this study, we aimed to assess the occurrence of bacterial pathogens associated with head lice collected from Niger’s refugees arriving in Algeria, using molecular tools. The determination of the genetic status of the head lice was also performed. Besides, a comparative sample of head lice collected from local population (non-migrant) was submitted to the same examination in the order to frame the results obtained within the head lice of Nigerien refugees (migrant population).

## Methods

### Louse sampling

**Fig. 1:**
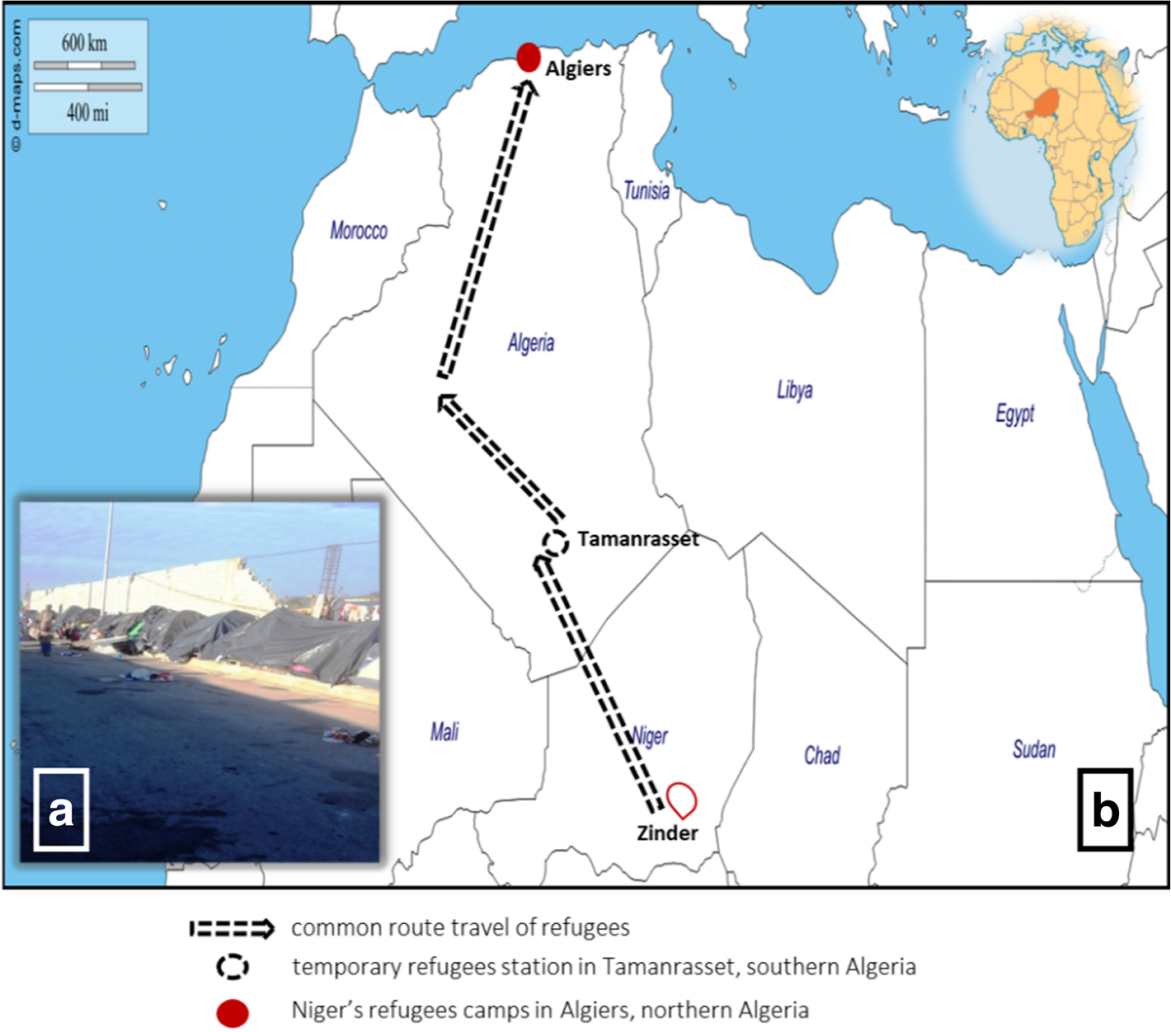
**a** Refugee camp showing squalor and unhygienic conditions. **b** Travel routes of Niger’s refugees from Zinder, Niger, western Africa to Algiers, Algeria, northern Africa

In January 2016, an epidemiological investigation was established in the Bab Ezzouar Nigerien refugees camp (36°43′00″N, 3°11′00″E), East Algiers, Algeria. Most refugees came from Zinder, southern Niger, after stopping in Tamanrasset, located in the extreme south of Algeria (Fig. 1). All of the sampled individuals were examined for the presence of both head and body lice; however, no body lice were found during the examination. A total of 37 head lice were collected from 31 individuals.

**Fig. 2:**
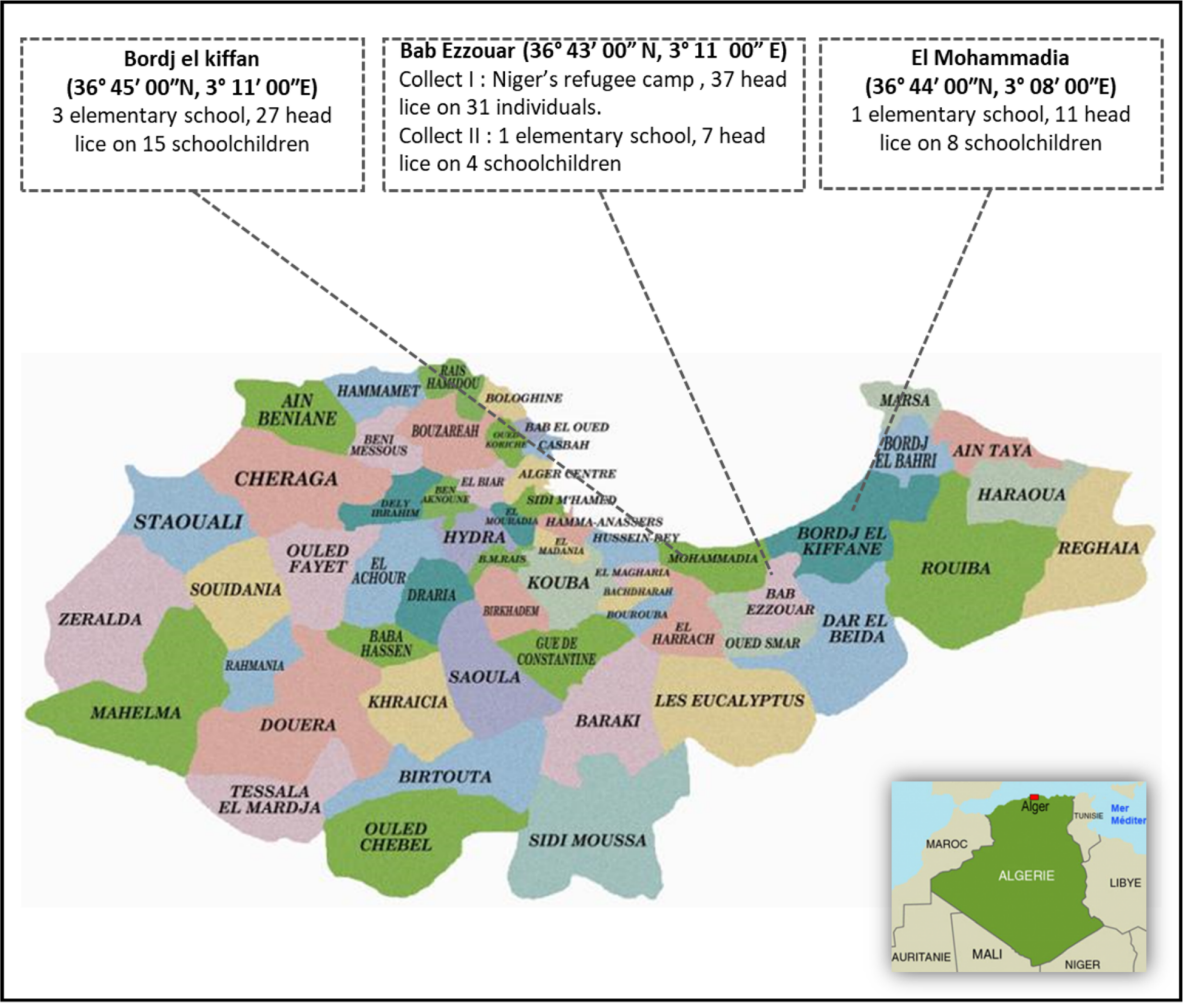
Map of head lice collection on Niger’s refugee (migrant population) and elementary schoolchildren (non-migrant population) from three localities in Algiers, Algeria

Between November 2015 and May 2016, head lice were collected from schoolchildren in 5 elementary schools at 3 different location in eastern Algiers, north Algeria, including the regions of Bab Ezzouar, El Mohammadia (36°44′00″N, 3°08′00″E,) and Bordj el kiffan (36°45′00″N, 3°11′00″E), in order to have a comparative sample from the local population. A total of 45 head lice were collected from 27 schoolchildren (Fig. 2).

Visible lice were removed from scalps using clamps and were immediately frozen at -20 °C and then transported to the IHU Méditerrannée-Infection, Marseille. All head lice collected were then processed for molecular study.

### DNA extraction

Prior to DNA isolation, the surface of each louse was decontaminated to avoid external contamination, as described previously [33]. Each louse specimen was cut in half length-ways. DNA was extracted from one half and the remaining halves of the lice were frozen at -20 °C for subsequent studies. DNA was extracted using the QIAamp DNA tissue extraction kit (Qiagen, Hilden, Germany) on the BioRobot EZ1 (Qiagen, Courtaboeuf, France) following the manufacturer’s instructions. DNA was eluted in 100 μl of TE buffer and stored at -20 °C until the next investigation.

### Genotypic status of lice

To identify the mitochondrial clades and to perform a phylogenetic study of the lice collected, all the DNA samples were subjected to standard PCR, targeting a 347 bp fragment of the *cytb* gene. The reaction of amplification was conducted in a final volume of 50 μl, including 25 μl Amplitaq gold master mix, 1 μl of each primer, 5 μl of DNA template, and water. The thermal cycling condition was as follows: an initial denaturation step at 95 °C for 15 min, followed by 40 cycles consisting of 1 min denaturation at 95 °C, 30 s annealing at 55 °C and a 1 min extension at 72 °C. A final extension cycle at 72 °C for 7 min was performed and the reactions were cooled at 15 °C. PCR amplification was performed in a Peltier PTC-200 model thermal cycler (MJ Research Inc., Watertown, MA, USA). Successful amplifications were confirmed via electrophoresis on 1.5% agarose gel. Purification of PCR products was performed using NucleoFast 96 PCR plates (Macherey Nagel EURL, Hoerdt, France) following the manufacturer’s instructions. The amplicons were sequenced using the Big Dye Terminator Cycle Sequencing Kit (Perkin Elmer Applied Biosystems, Foster City, CA) with an ABI automated sequencer (Applied Biosystems). The electropherograms obtained were then assembled and edited using ChromasPro software (ChromasPro 1.7, Technelysium Pty Ltd., Tewantin, Australia) and compared with those available in the GenBank database by NCBI BLAST (http://blast.ncbi.nlm.nih.gov/Blast.cgi).

### Molecular screening for the presence of pathogens

The DNA of all head lice were subject to amplification by qPCR using primers and probes targeting different genes of *Rickettsia* spp., *Borrelia* spp., *B. quintana*, *Y. pestis*, *Acinetobacter* spp., *C. burnetii* and *Anaplasma* spp., as previously reported (Table 1). All qPCRs were performed using a CFX96 Real-Time system (Bio-Rad, Marnes-la-Coquette, France) and the Eurogentec Master Mix Probe PCR kit (Eurogentec, Liège, Belgium). We included target bacterial DNA as the positive control and master mixtures as a negative control for each test. Samples were considered positive when the cycle threshold (Ct) was lower than 35 Ct. All *C. burnetii* positives samples were confirmed by a second specific qPCR targeting the IS30A spacer (Table 1).

**Table 1:** Oligonucleotide sequences of primers and probes used for real-time PCRs and conventional PCRs in this study

Target	Name	Primers (5′-3′) and probes	Source
*Pediculus humanus cytochrome b*	*Cytb*	F_GAGCGACTGTAATTACTAATC	[53]
		R_CAACAAAATTATCCGGGTCC	
*Acinetobacter* spp*.* RNA polymerase β subunit gene	*rpoB*	F_TACTCATATACCGAAAAGAAACGG	[51]
		R_GGYTTACCAAGRCTATACTCAAC	
		FAM-CGCGAAGATATCGGTCTSCAAGC-TAMRA	
	*rpoB* (zone1)	F_TAYCGYAAAGAYTTGAAAGAAG	[54]
		R_CMACACCYTTGTTMCCRTGA	
*Rickettsia* spp. citrate synthase (*gltA*)	RKNDO3	F_AATGCTCTTGCAGCTGGTTCT	[55]
		R_TCGAGTGCTAATATTTTTGAAGCA	
		FAM-CGGTGGTGTTAATGCTGCGTTACAACA-TAMRA	
*Yersinia pestis* plasminogen activator gene	*PLA*	F_ATGGAGCTTATACCGGAAAC	[56]
		R_GCGATACTGGCCTGCAAG	
		FAM-TCCCGAAAGGAGTGCGGGTAATAGG-TAMRA	
*Borrelia* spp. *16S ribosomal RNA*	Bor16S	F_AGCCTTTAAAGCTTCGCTTGTAG	[57]
		R_GCCTCCCGTAGGAGTCTGG	
		FAM-CCGGCCTGAGAGGGTGAACGG-TAMRA	
*Bartonella quintana* Hypothetical intracellular effector 3-oxoacyl-synthase gene	*yopP*	F_TAAACCTCGGGGGAAGCAGA	[58]
		R_TTTCGTCCTCAACCCCATCA	
		FAM-CGTTGCCGACAAGACGTCCTTG-TAMRA	
	*fabF3*	F_GCGGCCTTGCTCTTGATGA	
		R_GCTACTCTGCGTGCCTTGGA	
		FAM-TGCAGCAGGTGGAGAGAACGTG-TAMRA	
*Anaplasma* spp. 23S ribosomal RNA	*TtAna*	F_TGACAGCGTACCTTTTGCAT	[59]
		R_TGGAGGACCGAACCTGTTAC	
		FAM-GGATTAGACCCGAAACCAAG-TAMRA	
*Coxiella burnetii* Spacers	IS1111	F_CAAGAAACGTATCGCTGTGGC	[42]
		R_CACAGAGCCACCGTATGAATC	
		FAM-CCGAGTTCGAAACAATGAGGGCTG-TAMRA	
	IS30A	F_ CGCTGACCTACAGAAATATGTCC	
		R_ GGGGTAAGTAAATAATACCTTCTGG	
		FAM-CATGAAGCGATTTATCAATACGTGTATGC-TAMRA	
	Cox2	F_CAACCCTGAATACCCAAGGA	[34]
		R_GAAGCTTCTGATAGGCGGGA	
	Cox5	F_CAGGAGCAAGCTTGAATGCG	
		R_TGGTATGACAACCCGTCATG	
	Cox18	F_CGCAGACGAATTAGCCAATC	
		R_TTCGATGATCCGATGGCCTT	
	Cox22	F_GGGAATAAGAGAGTTAGCTCA	
		R_CGCAAATTTCGGCACAGACC	

In order to perform genotyping of *C. burnetii*, all positive samples were subjected to PCR amplification of the multi-spacer typing (MST), targeting four intergenic spacers (Cox2, Cox5, Cox18 and Cox22), as described previously [34]. To identify the species of the *Acinetobacter* bacteria, all samples tested positive by qPCRs for *Acinetobacter* spp. were subjected to standard PCR targeting a 350 bp fragment of the *rpoB* gene (zone1) (Table 1). Negative and positive controls were included in each assay. Successful amplification was confirmed via gel electrophoresis and amplicons were prepared and sequenced using similar methods as described for the *cytb* gene for lice above.

### Data analysis

The head lice nucleotide sequences obtained in this study were combined with the *cytb* database, comprising haplotypes that span different geographical location in the five continents, as previously reported [9]*.* MEGA 6.06 was used for the phylogenetic analyses under the Kimura 2-parameter model with 500 replicates, as described previously [35]. All obtained sequences of *Acinetobacter* species and *C. burnetii* were analyzed using BLAST (www.ncbi.nlm.nih.gov/blast/Blast.cgi) and compared to sequences in the GenBank database. Phylogenetic analyses using similar methods, as described above, were used to determine the position of *Acinetobacter* species identified in head lice of the two population compared to another *Acinetobacter* available in the GenBank database.

## Results

### Lice clade and phylogenetic analysis

Regarding the migrant population, we examined, in total, fifty female (≥ 24 years-old) and twenty child refugees (≤ 5 years-old). Twenty-eight of the fifty women (56%) and three of the 20 (15%) children were infested with head lice and were included in this study. Thirty-seven head lice were collected from 31 individuals refugees from the Nigerien refugee camp located in eastern Algiers, Algeria.

Fourty-five head lice specimens were collected from the non-migrant population. The sampling was performed in 5 elementary schools at 3 different locations in east Algiers, north Algeria. Of the 101 schholchildren examined, 27 (26.73%) were infested by head lice and the totality were female aged between 6–11 years-old. The highest infestation rate was recorded in the Bordj El kiffan region with a rate of 48.14% (13/27), followed by the Bab Ezzouar and El Mohammadia regions, with a rate of 33.33% (9/27) and 18.51% (5/27), respectively.

**Fig. 3:**
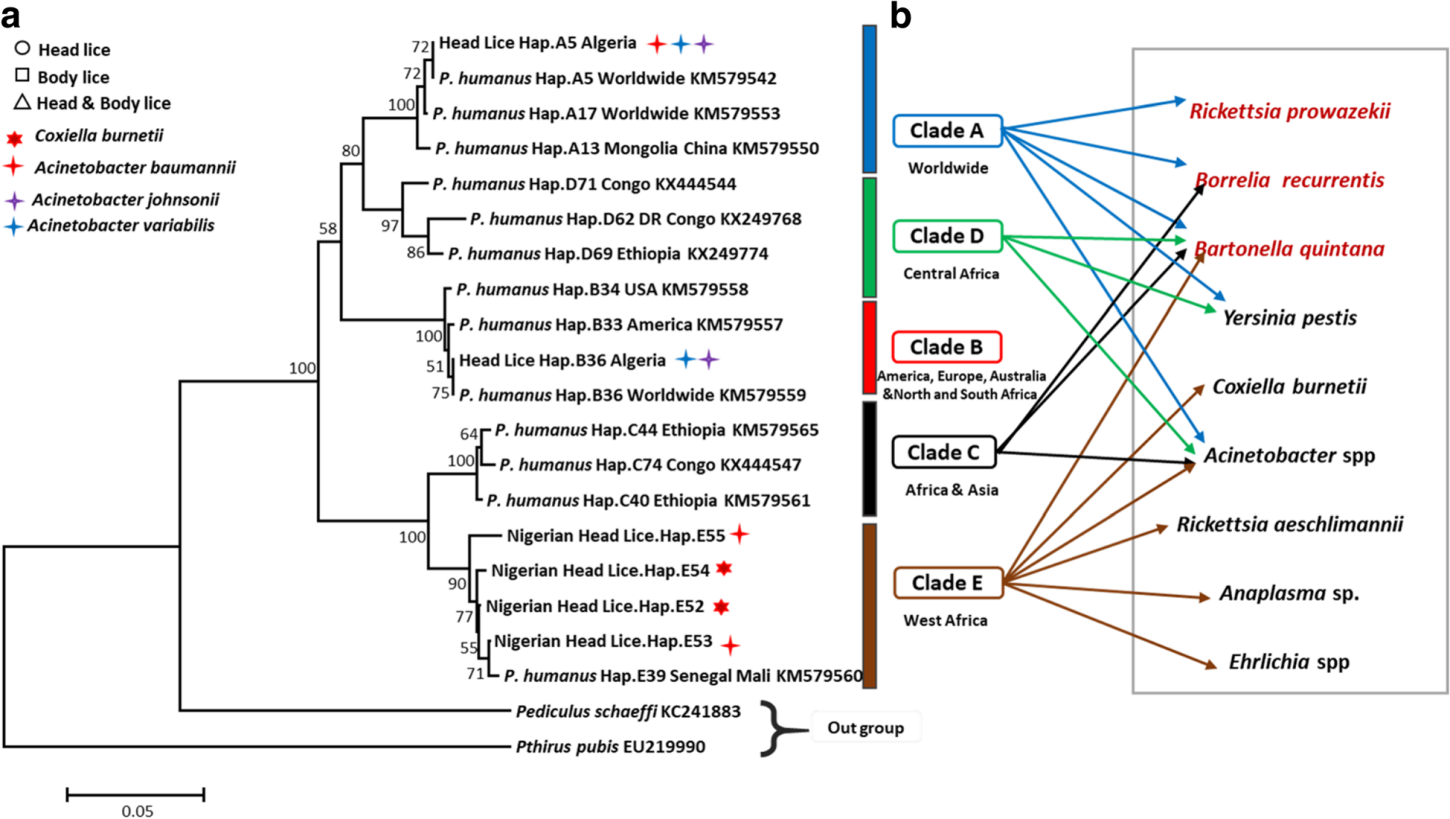
Maximum likelihood (ML) phylogram of the mitochondrial cytochrome *b* (*cytb*) gene. Phylogenetic inferences were conducted in MEGA 7 using the maximum likelihood method based on the Kimura 2-parameter. The mitochondrial clade memberships are indicated to the right of each tree. **a** Lice samples which are positive for *Coxiella burnetii* and *Acinetobacter spp.* and their genotypes (head or body lice) are specified (see legends at the top left). **b** Bacterial DNA detected in head lice reported in this study and the literature. The pathogenic bacteria in red are those naturally transmitted by body lice to humans

In total, 82 head lice samples were collected from Nigerien refugees and schoolchildren in eastern Algiers, and were then analyzed using PCR standard targeting the *cytb* gene to determine their clade. Results showed that all head lice of the migrant population (37/82, 45.12%) were clade E, and defined the presence of four different new haplotypes referred here as E52, E53, E54 and E55 (26/37, 70.27%) lice belonged to haplotype E52 (5/37, 13.51%) to haplotype E53 (4/37, 10.81%) to haplotype E54 and (2/37, 5.40%) to haplotype E55. While the head lice of the non-migrant population were clade A and B, the analysis of the 45 *cytb* sequences yielded 34/82 lice (42.68%) belonged to the worldwide haplotype A5 within the clade A and 11/82 (13.41%) belonged to the B36 haplotype, the most widespread and prevalent within the B haplogroup (Table 2). These haplotypes, together with references from all the head lice and haplogroups, were used to construct a maximum likelihood (ML) phylogenetic tree (Fig. 3).

**Table 2:** Detection of head lice clades and pathogens in the Nigerien refugees (migrant population) and schoolchildren (non-migrant population) in eastern Algiers, Algeria

Location	Population	No. lice tested (%)	Clade of lice (no.) %	Haplotype (no.) %	Pathogen (no.) %
Bab Ezzouar	Nigerien refugees	37 (45.12)	E (37/37) 100	E52: (26/37) 70.27	*Coxiella burnetii* (3/37) 8.10
				E53: (5/37) 13.51	*Acinetobacter baumannii* (20/37) 54.05
				E54: (4/37) 10.81	
				E55: (2/37) 5.40	
	Schoolchildren	7 (8.53)	A (3/7) 42.85	A5 (3/7) 42.85	*Acinetobacter johnsonii* (11/45) 24.44
			B (4/7) 57.14	B36 (4/7) 57.14	*Acinetobacter variabilis* (3/45) 6.66
El Mohammadia	Schoolchildren	11 (13.41)	A (9/11) 81.81	A5 (9/11) 81.81	*Acinetobacter johnsonii* (7/45) 15.55
			B (2/11) 18.18	B36 (2/11) 18.18	*Acinetobacter variabilis* (7/45) 15.55
					*Acinetobacter baumannii* (4/45) 8.88
Bordj El kiffan	Schoolchildren	27 (32.92)	A (20/27) 74.07	A5 (20/27) 74.07	*Acinetobacter johnsonii* (6/45) 13.33
			B (7/27) 25.92	B36 (7/27) 25.92	*Acinetobacter variabilis* (2/45) 4.44
Total	2	82 (100)	A (34/82) 41.46	E52: (26/37) 70.27	*Coxiella burnetii* (3/82) 3.65
			B (11/82) 13.41	E53: (5/37) 13.51	*Acinetobacter baumannii* (29/82) 35.36
			E (37/82) 45.12	E54: (4/37) 10.81	*Acinetobacter johnsonii* (24/82) 29.26
				E55: (2/37) 5.40	*Acinetobacter variabilis* (12/82) 14.63
				A5 (34/82) 41.46	
				B36 (11/82) 13.41

### Molecular detection of bacterial pathogens

In this study, the qPCR investigation of all lice samples for *Rickettsia* spp., *Borrelia* spp., *Y. pestis*, *B. quintana* and *Anaplasma* spp. produced no positive results. However, positive results were obtained when testing for the presence of *C. burnetii* and *Acinetobacter* spp.

The DNA of *C. burnetii* was detected in 3 out of 37 (8.10%) of head lice of Nigerien refugees collected from 3 of the 28 (10.71%) infested women but not reported in head lice of schoolchildren. The positive lice for *C. burnetii* belonged to haplotypes E52 and E54 (Fig. 3) (Table 2). These results were confirmed by targeting two C. *burnetii*-specific genes using qPCR, supplemented by PCR-sequencing of one spacer for the genotyping of *C. burnetii*. We only succeeded in obtaining sequences for the Cox22 spacer, probably due to the low concentration of *C. burnetii* DNA in these head lice samples. Obtained sequences did not allow to identify the *C. burnetii* genotype, but it corresponds to one of the eight possible MST genotypes of *C. burnetii*: 8, 9, 10, 38, 43, 48, 50 and 53. Negative controls remained negative in all PCR experiments.

**Fig. 4:**
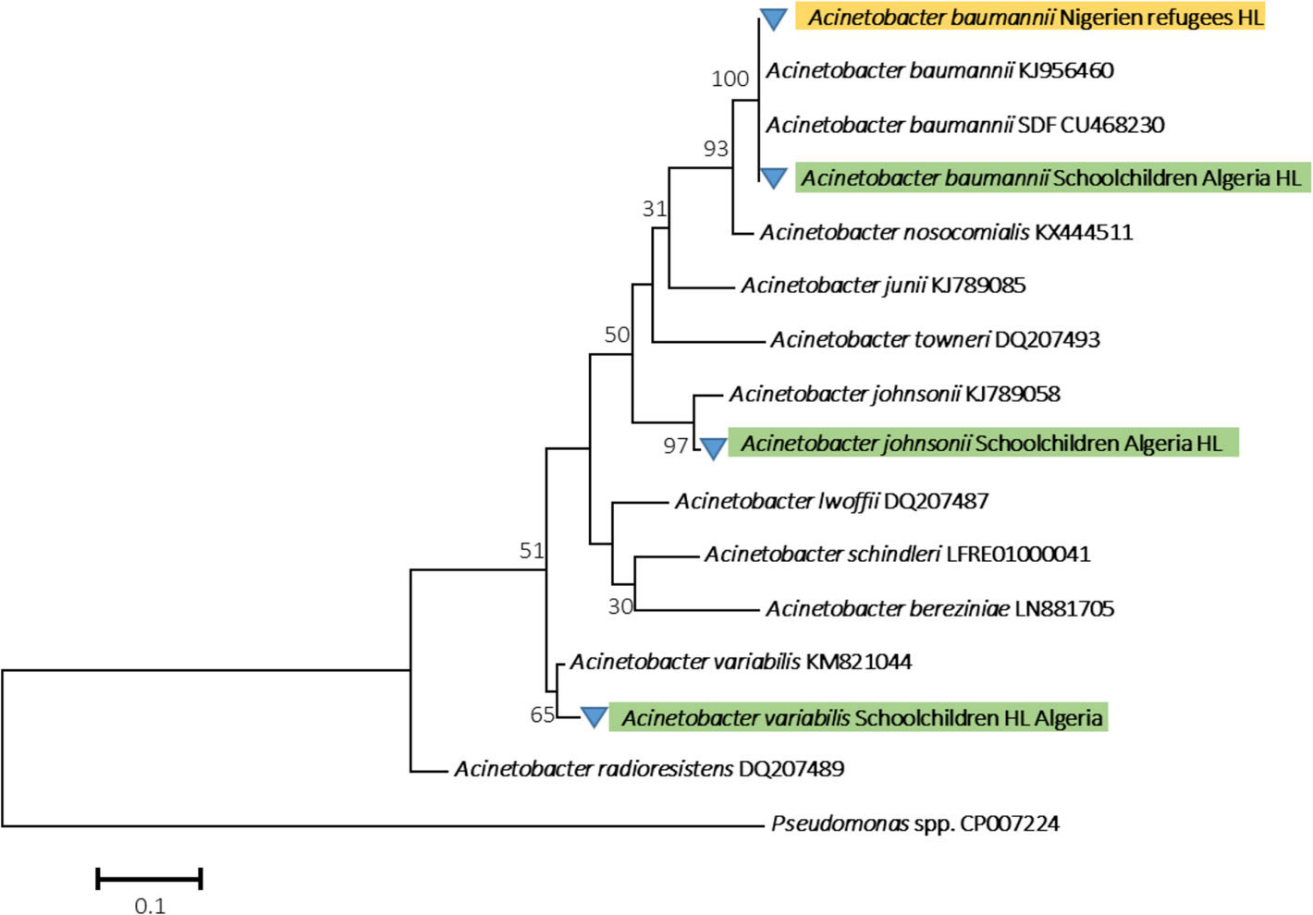
Phylogenetic tree highlighting the position of the *Acinetobacter* species identified in head lice of Nigerien refuges and schoolchildren compared to another *Acinetobacter* available in the GenBank database. Phylogenetic inferences were conducted in MEGA 7 using the maximum likelihood method based on the Kimura 2-parameter model for nucleotide sequences. Statistical support for internal branches of the tree was evaluated by bootstrapping with 500 iterations

The DNA of *Acinetobacter* spp. was detected in 20 out of 37 (54.05%) head lice collected from 25 out of 31 (80.64%) Nigerien refugees and in 40 out of 45 (88.88%) head lice collected from 15 out of 27 (55.55%) schoolchildren targeting the *rpob* gene using qPCR. As for the molecular identification of the *Acinetobacter* species, we succeeded in amplifying a 350 bp fragment of the *rpoB* (zone1) gene in all head lice positive in qPCR collected from the two populations (migrant and non-migrant). Based on a BLAST search, the comparison of the nucleotide sequences with the GenBank database sequences revealed the existence of one species of *Acinetobacter* for the head lice of Niger’s refugees sharing 99–100% identity with their corresponding references and identified as *A. baumannii*. All lice belonged to the four haplotypes of clade E found in this study (Fig. 3) (Table 2). DNA of *Acinetobacter* spp. of head lice collected from schoolchildren were identified: 24/40 (60%) positive-head lice as *A. johnsonii*, 12/40 (30%) sequences as *A. variabilis* and 4/40 (10%) sequences as *A. baumannii*, sharing 99–100% identity with their corresponding references and all belonged to the two haplogroup A and B (Fig. 4).

## Discussion

In recent years, several countries around the world and particularly those in the Horn of Africa, have endured humanitarian crisis conditions, including civil wars. The European Union has received thousands of refugees and migrants, representing a risk factor for possible outbreaks of arthropod-borne diseases. The current refugee crisis in Europe has been characterized by the rapid emergence of several cases of louse-borne relapsing fever caused by *B. recurrentis* which were diagnosed in the Netherlands**,** Germany, southern and northern Italy, Belgium, Switzerland and Finland [28–32].

Algeria is one of the countries in north Africa that has also received thousands of refugees and migrants from different countries, including Syria, Libya, Mali and Niger. An epidemiological survey was therefore established in Algiers at a camp of Nigerien refugees that housed more than a hundred individuals crowded together surviving in precarious conditions. These refugees have traveled thousands kilometers to reach their target country, providing thus an ideal environment for the spread of lice and the pathogens that they might carry.

Our study is the first to assess the genetic diversity as well as the occurrence of bacterial pathogens from head lice collected in camp of Nigerien refugees arriving from Zinder in Algeria, northern Africa. In this work, the pediculosis patients were female, 90.32% (28/31) were adult and 9.67% (3/31) were girls. As mentioned earlier, we performed the same analysis for head lice specimens collected from schoolchildren of local population in the order to have comparative sample to frame the results obtained in this study.

The mitochondrial DNA analysis of the 37 head lice of the migrant population, and the 45 head lice of the non-migrant population, showed the presence of three major haplogroups: A, B and E. All Niger’s refugees head lice tested (37/82) belonged to clade E, including four different new haplotypes characterized in this study: haplotype E52 was the most prevalent (70.27%), followed by haplotype E53 (13.51%), haplotype E54 (10.81%) and, finally, haplotype E55 (5.40%) (Table 2). Previous studies have already reported that clade E is limited to west African countries, namely Senegal and Mali [9]. It was then recently reported, for the first time, in head lice from Bobigny (France), also sampled from families originating from west African countries [16]. The remaining head lice samples collected from sccholchildren in eastern Algiers, belonged to the worldwide haplotype A5 (34/82, 42.68%) and the most widespread haplotype B6 (11/82, 13.41%), within the clade A and B, respectively. These data confirm the existence of clade A and B in Algeria, as reported by previous studies [36, 37, 27].

This is the first report of clade E among head lice of refugees coming from Niger, west Africa, to Algeria, assuming that the head-louse infestation occurred in their country of origin, and that clade E may be a vector for human pathogens. These results are not surprising, as expected, they confirm that clade E has a west African distribution, as reported by previous studies [9, 16].

To date, human lice infestation remains a global public health and social problem [38]. Although body lice are assumed to be the main vectors of pathogens, the epidemiological status of head lice as a vector of louse-borne diseases is still misunderstood. Even though, it has been demonstrated that the immune reactions of head lice to different pathogens are stronger than those of the body louse, which may enable it to carry a large spectrum of pathogens [39, 40]. In natural settings, head lice belonging to different mitochondrial clades sampled from several countries have been found to carry the DNA of several pathogen bacteria including *B. quintana*, *B. recurrentis*, *B. theileri*, *Acinetobacter* spp. and *Y. pestis*, as well as the DNA of *C. burnetii*, *R. aeschlimannii* and two potential new species of the genera *Anaplasma* and *Ehrlichia*, of unknown pathogenicity [11, 14, 16, 19, 21–27]. Experimental studies have demonstrated that head lice may also act as a vector for louse-borne diseases [33, 41].

*Coxiella burnetii,* the causative agent of Q fever, is a highly infectious zoonotic intracellular bacterium. It is found worldwide and has a diverse wide range of hosts including mammals, birds, reptiles and arthropods, mainly ticks [42]. The infection is generally transmitted to humans through direct contact (milk, urine, feces or semen from infected animals) as well as through aerosol inhalation. It can be acute or chronic, exhibiting a wide range of clinical manifestations [42]. Q fever has been reported throughout the African continent with a higher prevalence in western Africa, including several countries such as Nigeria, Senegal, Ghana, Republic of Côte d’Ivoire, Burkina Faso and Niger, representing a significant public health threat [43]. In Niger, only one serological study has so far been carried out on humans, in Niamey, where 10% (17/177) of children aged between one month and five years-old were found to be seropositive for the *C. burnetii* infection [44]. Another study was conducted on the prevalence of *C. burnetii* in animals of the Maradi Region of Niger and showed that 32% (24/75) of goats that had experienced abortions were seropositive, compared to 29% (12/75) of non-randomly selected goats without a history of abortion [45].

We found 8.10% of 37 head lice infected by *C*. *burnetii* collected from three of 28 persons. This bacterium was not reported in head lice sampled from the non-migrant population. *Coxiella burnetii* was recently reported in 1% of 600 head lice belonging to clade E infesting 5% of 117 individuals from Mali [21]. In contrast, the presence of this bacterium was also investigated in Ethiopia on head and body lice, and results showed no evidence of *C. burnetii* in any of the specimens tested [46]. A field study in East Africa, showed that lice collected from individuals, living in a place where an epidemic of Q fever had occurred three months previously, could be spontaneously infected with *C. burnetii* [47]. Another study also showed that, under experimental conditions, it is possible to infect body lice with *C*. *burnetii*, although human lice are not a known vector of this bacterium [48]. The presence of *C. burnetii* has never been reported in head lice belonging to the other clades; however, it has been recently reported for the firt time in 10/524 (1.90%) body lice belonging to clade A collected from 2/19 (10.52%) homeless people in northern Algeria [49]. Based on our results and data from the literature, the role of human lice in the epidemiology of the *C. burnetii* infection should be further investigated.

This study also reveals the presence of *A*. *baumannii* DNA in Nigerien refugees head lice, and the presence of *A. johnsonii* and *A. variabilis* in addition to *A. baumannii* in head lice sampled from sccholchildren in eastern Algiers, Algeria. The DNA of *A. johnsonii* and *A. variabilis* was reported in head lice collected from all three localities of the collection, while DNA of *A. baumannii* was reported only in El Mohamammadia. Findings from previous study on head lice collected from elementary schoolchildren in four localities in Algiers, Algeria have led to the same results, whither a widespreading infection of head lice with the same *Acinetobacter* species found in our study [27].

*Acinetobacter baumannii* was isolated for the first time from the body lice found on homeless people in Marseille (France), as well as from various countries around the world [50]. In recent years, the DNA of *A*. *baumannii*, in addition to several species of *Acinetobacter*, were found in head lice collected from elementary schoolchildren in Paris belonging to clade A [51], in Thailand belonging to A and C [14], in Algeria belonging to clade A [27] and in head lice collected from pygmy populations in the Republic of the Congo belonging to clades A, D, and C [11]. *Acinetobacter baumannii* DNA has also been detected in head lice collected from healthy individuals in Ethiopia [26]. The presence of *A. baumannii* DNA was even identified in ancient human head lice remains belonging to clade A [9]. Our study is the first to report the identification of *A. baumannii* DNA in Nigerien head lice belonging to clade E, as well as the identification of several species of *Acinetobacter* in head lice belonging to clade B in Algeria.

*Acinetobacter* species are widespread in nature, including in water, soil, living organisms and on the skin of patients and healthy subjects [52]. Recent works have shown that the *Acinetobacter* infection can be highly prevalent among body and head lice. However, it is still not clear whether these *Acinetobacter* strains, present in head lice, are the same as those responsible for human infections [26], the clinical significance of this finding is still unknown. Furthermore, molecular evidence for the presence of DNA of *C. burnetii* and several *Acinetobacter* species cannot distinguish between pathogens accidentally acquired from the blood of infected individuals, and those established in a competent vector which can maintain and transmit the pathogen [17].

## Conclusions

Our study is the first to report that head lice from Nigerian refugees belong to haplogroup E, and to confirm that the clade E has a west African distribution. We also detected, for the first time, the presence of *C. burnetii* and *A. baumannii* in head lice from Niger. Nevertheless, further studies of louse-borne pathogens are necessary to better understand the specificity of lice to different pathogenic bacteria. Further studies are also required in order to explain their ability to harbor and, in certain cases, transmit pathogens from one person to another. Moreover, these results indicate that refugee populations generate a potential risk of spreading emerging diseases and thus pose a significant epidemic threat to public health in Algeria, where individual refugees living in conditions of poverty and promiscuity may serve as indigenous reservoirs of multiple micro-organisms.

## Abbreviations

*Cyt b*: Cytochrome b
MST: Multispacer spacer typing
PCR: Polymerase chain reaction
qPCR: Quantitative real-time polymerase chain reaction
